# Exploring the relationships between heritage tourism, sustainable community development and host communities’ health and wellbeing: A systematic review

**DOI:** 10.1371/journal.pone.0282319

**Published:** 2023-03-29

**Authors:** Cristy Brooks, Emma Waterton, Hayley Saul, Andre Renzaho

**Affiliations:** 1 Translational Health Research Institute, School of Medicine, Western Sydney University, Sydney, Australia; 2 School of Social Sciences, Western Sydney University, Sydney, Australia; 3 Department of Archaeology, University of York, York, United Kingdom; 4 Maternal, Child and Adolescent Health Program, Burnet Institute, Melbourne, Australia; The Hong Kong Polytechnic University, HONG KONG

## Abstract

Previous studies examining the impact of heritage tourism have focused on specific ecological, economic, political, or cultural impacts. Research focused on the extent to which heritage tourism fosters host communities’ participation and enhances their capacity to flourish and support long-term health and wellbeing is lacking. This systematic review assessed the impact of heritage tourism on sustainable community development, as well as the health and wellbeing of local communities. Studies were included if they: (i) were conducted in English; (ii) were published between January 2000 and March 2021; (iii) used qualitative and/or quantitative methods; (iv) analysed the impact of heritage tourism on sustainable community development and/or the health and wellbeing of local host communities; and (v) had a full-text copy available. The search identified 5292 articles, of which 102 articles met the inclusion criteria. The included studies covering six WHO regions (Western Pacific, African, Americas, South-East Asia, European, Eastern Mediterranean, and multiple regions). These studies show that heritage tourism had positive and negative impacts on social determinants of health. Positive impacts included economic gains, rejuvenation of culture, infrastructure development, and improved social services. However, heritage tourism also had deleterious effects on health, such as restrictions placed on local community participation and access to land, loss of livelihood, relocation and/or fragmentation of communities, increased outmigration, increases in crime, and erosion of culture. Thus, while heritage tourism may be a poverty-reducing strategy, its success depends on the inclusion of host communities in heritage tourism governance, decision-making processes, and access to resources and programs. Future policymakers are encouraged to adopt a holistic view of benefits along with detriments to sustainable heritage tourism development. Additional research should consider the health and wellbeing of local community groups engaged in heritage tourism. Protocol PROSPERO registration number: CRD42018114681.

## Introduction

Tourism, heritage, and sustainable development go hand in hand. Socio-economically, tourism is considered a vital means of sustainable human development worldwide, and remains one of the world’s top creators of employment and a lead income-generator, particularly for Global South countries [1]. For most low- and middle-income countries (LMICs), tourism is a key component of export earnings and export diversification, and a major source of foreign-currency income [1]. In 2019, prior to the international travel restrictions implemented to contain the spread of coronavirus disease (COVID-19), export revenues from international tourism were estimated at USD 1.7 trillion, the world’s third largest export category after fuels and chemicals with great economic impacts. Tourism remains a major part of gross domestic product, generating millions of direct and indirect jobs, and helping LMICs reduce trade deficits [1]. It accounts for 28 per cent of the world’s trade in services, 7 per cent of overall exports of goods and services and 1 out of 10 jobs in the world [1]. Given this, it is anticipated that tourism will play a strong role in achieving all of the Sustainable Development Goals (SDGs), but particularly Goals 1 (No poverty), 8 (Decent work and economic growth), 12 (Responsible consumption and production), 13 (Climate action) and 14 (Life below water).

To ensure tourism’s continued contribution to sustainable development efforts, the World Tourism Organisation (UNWTO) has established the T4SDG platform in order to “to make tourism matter on the journey to 2030” [2]. Likewise, in recognition of the relationship between heritage, tourism, and sustainable development, UNESCO launched the World Heritage and Sustainable Tourism Programme, which was adopted by the World Heritage Committee in 2012. This Programme encapsulates a framework that builds on dialogue and stakeholder cooperation to promote an integrated approach to planning for tourism and heritage management in host countries, to protect and value natural and cultural assets, and develop appropriate and sustainable tourism pathways [3].

The addition of ‘heritage’ creates an important sub-category within the tourism industry: heritage tourism. This study adopts a broad definition of ‘heritage’, which encompasses the intersecting forms of *tangible* heritage, such as buildings, monuments, and works of art, *intangible or living* heritage, including folklore, cultural memories, celebrations and traditions, and *natural* heritage, or culturally infused landscapes and places of significant biodiversity [4]. This encompassing definition captures ‘heritage’ as it is understood at the international level, as evidenced by two key UNESCO conventions: the 1972 *Convention Concerning the Protection of the World Cultural and Natural Heritage*, which protects cultural, natural, and mixed heritage; and the 2003 *Convention for the Safeguarding of the Intangible Cultural Heritage*, which protects intangible heritage. Although the identification, conservation and management of heritage has traditionally been driven by national aspirations to preserve connections with history, ancestry, and national identity, the social and economic benefits of heritage tourism at community levels have also been documented [5].

Heritage tourism, as one of the oldest practices of travelling for leisure, is a significant sector of the tourism industry. It refers to the practice of visiting places because of their connections to cultural, natural, and intangible heritage and is oriented towards showcasing notable relationships to a shared past at a given tourism destination [4]. It contributes to global interchange and inter-cultural understanding [4]. Heritage tourism places economic and political value on recognised heritage resources and assets, providing additional reasons to conserve heritage further to the cultural imperatives for its maintenance [5]. By drawing on the cultural and historical capital of a community, heritage tourism can contribute to the flourishing of local communities and their *positive* sustainable development. However, as this systematic review will demonstrate, when applied uncritically and without meaningful engagement with the needs of local stakeholder, heritage tourism can also elicit damaging effects on community health and wellbeing.

First published in 1987, the classic report ‘*Our Common Future’*, more commonly known as the Brundtland Report, conceptualised sustainable development as “development that meets the needs of the present without compromising the ability of future generations to meet their own needs” [6]. Although this definition still works for many purposes, it emphasised the critical issues of environment and development whilst turning on the undefined implications of the word ‘needs’. In the report, the concept of sustainable development thus left unspecified the assumed importance of distinct cultural, political, economic, and ecological needs as well as health needs. Drawing on the work of globalization and cultural diversity scholar, Paul James [7], in this study we have defined ‘positive sustainable development’ as those “practices and meanings of human engagement that make for lifeworlds that project the ongoing probability of natural and social flourishing”, taking into account questions of vitality, relationality, productivity and sustainability.

### Study rationale

For many years, the impact of heritage tourism has predominantly been viewed through ecological [8, 9], economic and cultural [10, 11] or political [12] lenses. For example, it has often been assumed that the conservation of historic, cultural, and natural resources, in combination with tourism, will naturally lead to sustainable local economies through increases in employment opportunities, provisioning of a platform for profitable new business opportunities, investment in infrastructure, improving public utilities and transport infrastructures, supporting the protection of natural resources, and, more recently, improving quality of life for local residents [13–15].

Similarly, the impact of heritage tourism on health and wellbeing has tended to focus on visitors’ wellbeing, including their health education and possible health trends, medical aspects of travel preparation, and health problems in returning tourists [16–18]. It has only been more recently that host communities’ health needs and wellbeing have been recognised as an intrinsic part of cultural heritage management and sustainable community development [19]. In this literature, it has been hypothesised that potential health implications of heritage tourism are either indirect or direct. Indirect effects are predominantly associated with health gains from heritage tourism-related economic, environmental, socio-cultural, and political impacts [20]. In contrast, health implications associated with direct impacts are closely associated with immediate encounters between tourism and people [20]. Yet, little is known of the overall generative effects of heritage tourism on sustainable community development, or the long-term health and wellbeing of local communities. For the first time, this systematic review identified and evaluated 102 published and unpublished studies in order to assess the extent to which heritage tourism fosters host communities’ participation and, consequently, their capacity to flourish, with emphasis placed on the long-term health impacts of this. The primary objective of the review was to determine: (1) what the impacts of heritage tourism are on sustainable community development; as well as (2) on the health and wellbeing of local host communities. Understanding the relationship between heritage tourism, sustainable community development and health is essential in influencing policies aimed at improving overall livelihood in local host communities, as well as informing intervention strategies and knowledge advancement.

## Methods

This systematic review adhered to the guidelines and criteria set out in the Preferred Reporting Items for Systematic Reviews and Meta-Analyses (PRISMA) 2020 statement [21]. A protocol for this review was registered with PROSPERO (CRD42018114681) and has been published [22].

### Search strategy

In order to avoid replicating an already existing study on this topic, Cochrane library, Google Scholar and Scopus were searched to ensure there were no previous systematic reviews or meta-analyses on the impact of heritage tourism on sustainable community development and the health of local host communities. No such reviews or analyses were found. The search then sought to use a list of relevant text words and sub-headings of keywords and/or MeSH vocabulary according to each searched database. Derived from the above research question, the key search words were related to heritage tourism, sustainable community development, and health and wellbeing of local host communities. A trial search of our selected databases (see below) found that there are no MeSH words for heritage and tourism. Therefore, multiple keywords were included to identify relevant articles.

To obtain more focused and productive results, the keywords were linked using “AND” and “OR” and other relevant Boolean operators, where permitted by the databases. Subject heading truncations (*) were applied where appropriate. The search query was developed and tested in ProQuest Central on 22 November 2018. Following this search trial, the following combination of search terms and keywords, slightly modified to suit each database, was subsequently used:

(“Heritage tourism” OR tourism OR “world heritage site” OR ecotourism OR “heritage based tourism” OR “cultural tourism” OR “diaspora tourism” OR “cultural heritage tourism” OR “cultural resource management” OR “cultural heritage management” OR “historic site”)

AND

(“Health status” [MeSH] OR “health equity” OR health OR community health OR welfare OR wellbeing)

AND

(“sustainable development” [MeSH] OR sustainab* or “community development” or “local development” or “local community” or “indigenous community”)

The search covered the following bibliographic databases and electronic collections:

1. Academic Search Complete
2. Australian Heritage Bibliography (AHB)
3. Applied Social Sciences Index and Abstracts (ASSIA)
4. CAB Abstracts
5. CINAHL
6. EMBASE
7. PsycINFO
8. ProQuest Central
9. Science And Geography Education (SAGE)
10. Tourism, Hospitality and Leisure

In addition, grey literature were also sourced from key organisation websites including the International Union for Conservation of Nature (IUCN), the International Council on Monuments and Sites (ICOMOS), the International Centre for the Study of the Preservation and Restoration of Cultural Property (ICCROM), the International Centre for Integrated Mountain Development (ICIMOD), the International Council of Museums (ICOM), the United Nations Educational Scientific and Cultural Organization (UNESCO), the United Nations World Tourism Organisation (UNWTO) and the Smithsonian Institution.

Where the full texts of included articles could not be accessed, corresponding authors were contacted via e-mail or other means of communication (e.g., ResearchGate) to obtain a copy. A further search of the bibliographical references of all retrieved articles and articles’ citation tracking using Google Scholar was conducted to capture relevant articles that might have been missed during the initial search but that meet the inclusion criteria. For the purposes of transparency and accountability, a search log was kept and constantly updated to ensure that newly published articles were captured. To maximise the accuracy of the search, two researchers with extensive knowledge of heritage tourism literature (EW and HS) and two research assistants with backgrounds in public health and social sciences implemented independently the search syntax across the databases and organisations’ websites to ensure no article was missed.

### Inclusion and exclusion criteria

Criteria used in this systematic review focused on the types of beneficiaries of heritage tourism, outcomes of interest, as well as the intervention designs. The outcomes of interest were sustainable community development and evidence for the overall health and wellbeing of local host communities. In this systematic review, sustainable community development was defined in terms of its two components: ‘community sustainability’ and ‘development’. Community sustainability was conceptualised as the “long-term durability of a community as it negotiates changing practices and meanings across all the domains of culture, politics, economics and ecology” (pp. 21, 24) [23].

In contrast, development was conceptualised as “social change—with all its intended or unintended outcomes, good and bad—that brings about a significant and patterned shift in the technologies, techniques, infrastructure, and/or associated life-forms of a place or people” (p. 44) [7]. To this, we added the question of whether the development was positive or negative. Thus, going beyond the Brundtland definition introduced earlier and once again borrowing from the work of Paul James, positive sustainable development was defined as “practices and meanings of human engagement that make for lifeworlds that project the ongoing probability of natural and social flourishing”, including good health [23].

Health was defined, using the World Health Organisation (WHO) definition, as “overall well-being” and as including both physical, mental and social health [24]. While there is no consensus on what wellbeing actually means, there is a general agreement that wellbeing encompasses positive emotions and moods (e.g., contentment, happiness), the absence of negative emotions (e.g., depression, anxiety) as well as satisfaction with life and positive functioning [25]. Therefore, wellbeing in this systematic review was conceptualised according to Ryff’s multidimensional model of psychological wellbeing, which includes six factors: autonomy; self-acceptance, environmental mastery, positive relationships with others, purpose in life, and personal growth [26].

In terms of intervention and design, this systematic review included peer-reviewed and grey literature sources of evidence [27, 28] from quantitative, qualitative, and mixed methods studies. Intervention designs of interest were observational studies (e.g. longitudinal studies, case control and cross-sectional studies) as well as qualitative and mixed-methods studies. The following additional restrictions were used to ensure texts were included only if they were: (i) written in English; (ii) analysed the impact of heritage tourism on sustainable community development and health and/or wellbeing of local host communities; (iii) research papers, dissertations, books, book chapters, working papers, technical reports including project documents and evaluation reports, discussion papers, and conference papers; and (iv) published between January 2000 and March 2021. Studies were excluded if they were descriptive in nature and did not have community development or health and wellbeing indicators as outcome measures.

The year 2000 was selected as the baseline date due to the signing of the United Nations Millennium Development Goals (MDGs) by Member States in September of that year. With the introduction of the MDGs, now superseded by the Sustainable Development Goals (SDGs), there was an increase in commitment from government and non-governmental organizations to promote the development of responsible, sustainable and universally accessible tourism [29, 30]. Editorials, reviews, letter to editors, commentaries and opinion pieces were not considered. Where full text articles were not able to be retrieved despite exhausting all available methods (including contacting corresponding author/s), such studies were excluded from the review. Non-human studies were also excluded.

### Study selection and screening

**Fig 1:**
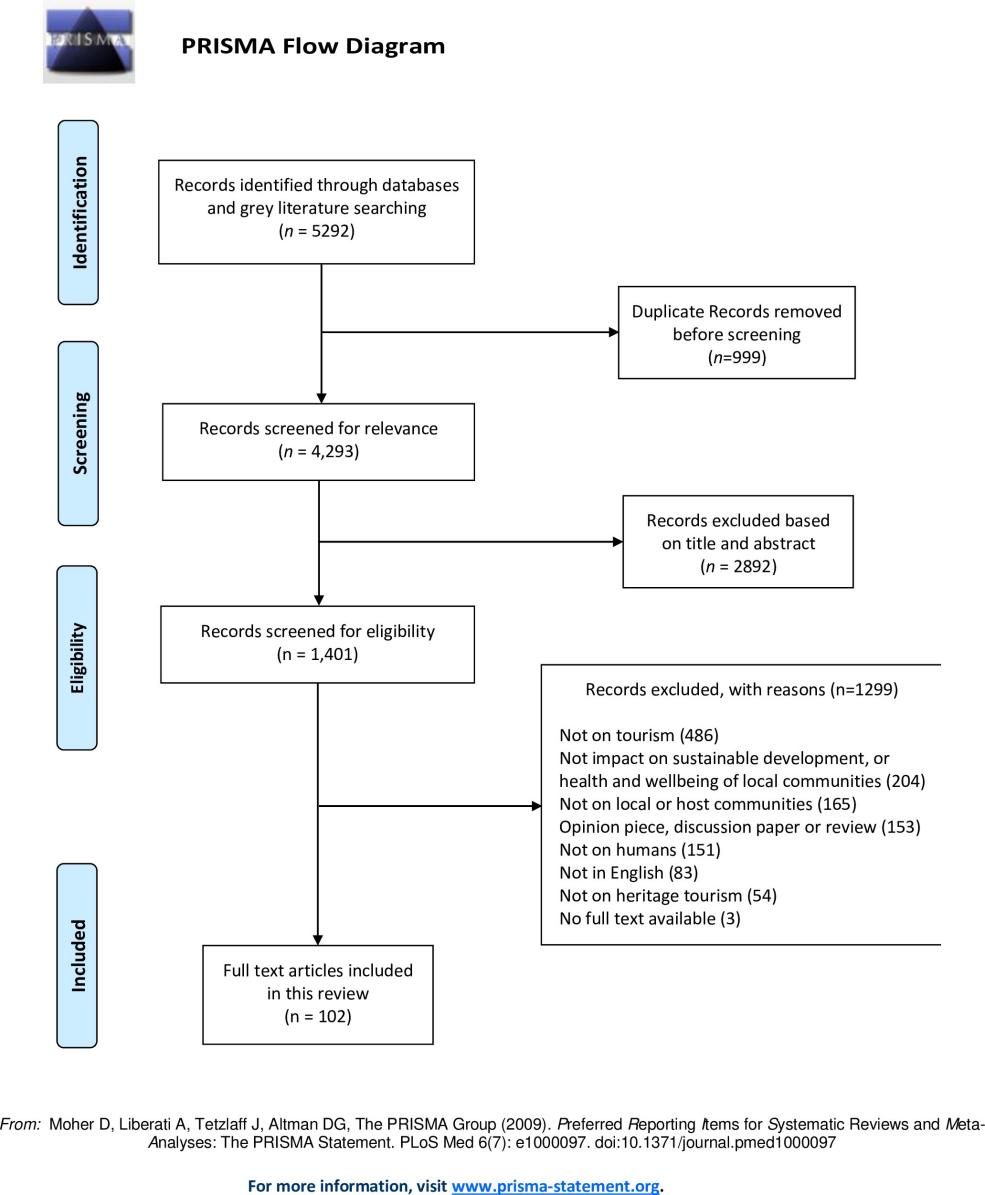
PRISMA diagram and flow chart.

Data retrieved from the various database searches were imported into an EndNote X9 library. A three-stage screening process was followed to assess each study’s eligibility for inclusion. In the EndNote library, stage one involved screening studies by titles to remove duplicates. In stage two, titles and abstracts were manually screened for eligibility and relevance. In the third and final screening stage, full texts of selected abstracts were further reviewed for eligibility. The full study selection process according to PRISMA is summarised in Fig 1. A total of 5292 articles from 10 databases and multiple sources of grey literature were screened. After removal of duplicates, 4293 articles were retained.

Titles and abstracts were further screened for indications that articles contain empirical research on the relationship between heritage tourism, sustainable community development and the health and wellbeing of local host communities. This element of the screening process resulted in the exclusion of 2892 articles. The remaining 1401 articles were screened for eligibility: 1299 articles were further excluded, resulting in 102 articles that met our inclusion criteria and were retained for analysis. Study selection was led by two researchers (EW and HS) and one research assistant, who independently double-checked 40% of randomly selected articles (n = 53). Interrater agreement was calculated using a 3-point ordinal scale, with the scoring being ’yes, definitely in’ = 1, ’?’ for unsure = 2, and ’no, definitely out’ = 3. Weighted Kappa coefficients were calculated using quadratic weights. Kappa statistics and percentage of agreement were 0.76 (95%CI: 0.63, 0.90) and 0.90 (95%CI: 0.85, 0.96) respectively, suggesting excellent agreement.

### Data extraction

Data extraction was completed using a piloted form and was performed and subsequently reviewed independently by three researchers (AR, EW and HS), all of whom are authors. The extracted data included: study details (author, year of publication, country of research), study aims and objectives, study characteristics and methodological approach (study design, sample size, outcome measures, intervention), major findings, and limitations.

### Quality assessment

To account for the diversity in design and dissemination strategies (peer-reviewed vs non-peer-reviewed) of included studies, the (JBI) Joanna Briggs Institute’s Critical Review Tool for qualitative and quantitative studies [31], mixed methods appraisal Tool (MMAT) for mixed methods [32], and the AACODS (Authority, Accuracy, Coverage, Objectivity, Date, Significance) checklist for grey literature [33] were used to assess the quality of included studies. The quality assessment of included studies was led by one researcher (CB), but 40% of the studies were randomly selected and scored by three senior researchers (AR, EM, and HS) to check the accuracy of the scoring. Cohen’s kappa statistic was used to assess the agreement between quality assessment scorers. Kappa statistics and percentage of agreement were 0.80 (95%CI: 0.64, 0.96) and 0.96 (95%CI: 0.93, 0.99) respectively, suggesting excellent interrater agreement. The quality assessment scales used different numbers of questions and different ranges, hence they were all rescaled/normalised to a 100 point scale, from 0 (poor quality) to 100 (high quality) using the min-max scaling approach. Scores were stratified by tertiles, being high quality (>75), moderate quality (50–74), or poor quality (<50).

### Data synthesis

Due to the heterogeneity and variation of the studies reviewed (study methods, measurements, and outcomes), a meta-analysis was not possible. Campbell and colleagues (2020) [34] recognise that not all data extracted for a systematic review are amenable to meta-analysis, but highlight a serious gap in the literature: the authors’ lack of or poor description of alternative synthesis methods. The authors described an array of alternative methods to meta-analysis. In our study we used a meta-ethnography approach to articulate the complex but diverse outcomes reported in included studies [35]. Increasingly common and influential [36], meta-ethnography is an explicitly interpretative approach to the synthesis of evidence [36, 37] that aims to develop new explanatory theories or conceptualisations of a given body of work on the basis of reviewer interpretation [37]. It draws out similarities and differences at the conceptual level between the findings of included studies [37], with the foundational premise being the juxtaposition and relative examination of ideas between study findings [37]. Resulting novel interpretations are then considered to transcend individual study findings [36].

Originating with sociologists Noblit and Hare [36, 38], and adopted and expanded upon by other researchers [36, 37], meta-ethnography involves a 7-stage process of evidence synthesis and concludes with the translation and synthesis of studies [38]. The approach centres around the emergence of concepts and themes from included studies that are examined in relation to each other and used to synthesise and communicate primary research findings. In meta-ethnography, the diversity of studies such as the heterogeneity and variation of included studies in the present review, is considered an asset opposed to an issue in synthesis or translation of research findings [37].

Common threads, themes and trends were identified and extracted from both qualitative and quantitative narratives to generate insight on the impact of heritage tourism on sustainable community development and health. In order to increase reproducibility and transparency of our methods and the conclusions drawn from the studies, the narrative synthesis adhered to the “Improving Conduct and Reporting of Narrative Synthesis of Quantitative Data” protocol for mixed methods studies [39]. One of the primary researchers (CB) summarised the study findings and narrated the emerging themes and subthemes. The emerging themes were discussed with all authors for appropriateness of the content as well as for consistency. All studies were included in the synthesis of evidence and emergence of themes. The meta-ethnographic approach involved the following processes:

#### Identifying metaphors and themes

Included studies were read and reviewed multiple times to gain familiarity and understanding with the data and identify themes and patterns in each study. As noted above, data was extracted from each study using a piloted template to remain consistent across all studies. The aims and/or objectives of each study was revisited regularly to validate any extracted data and remain familiar with the purpose of the study. Themes and, where relevant, sub-themes were identified, usually in the results and discussion section of included studies.

#### Determining how the studies were related

Studies were grouped according to WHO regions (see Table 1). Thematic analysis was compared across all included studies regardless of region to identify common themes and/or sub-themes to determine how studies were related to one another. Although this review included a widely varied and large number of studies (n = 102), the findings of each study nonetheless had a common underpinning theme of heritage-based tourism. This enabled the identification of communal categories across the studies indicating their relatedness. For example, there were common themes of socio-cultural, socio-economic, community health, wellbeing, and empowerment factors and so on.

**Table 1:** Descriptive characteristics and quality assessment for included studies by WHO Region.

WHO Region	Reference	Author	Year	Country	Aim	Design	Methods	Data Collection	Sustainability variables	Quality Rating
African	[40]	Mbaiwa	2004	Botswana	To evaluate the sociocultural impacts of tourism development in Okavango Delta, Botswana	Mixed methods	Survey and interviews	Survey was administered to 65 Safari Managers, 98 safari workers Interviews with government officials and community-based tourism organisations.	Social impacts Cultural impacts Ecological Economic impacts	Moderate
African	[41]	Melubo and Lovelock	2019	Tanzania	To examine perceptions of the Maasai community regarding the impacts of the World Heritage Site on their livelihood	Qualitative	Semi-structured interviews	112 semi-structured interviews with residents, traditional Maasai leaders, Village leaders and Pastoral Council officials	Economic Social	Poor
African	[42]	Eja and Effiom	2015	Nigeria	To assess the socioeconomic impact of the yam festival on local communities	Quantitative	Survey	750 randomly sampled people in five communities	Economic Social	Poor
African	[43]	Anderson	2015	Tanzania	To examine the perspectives of local communities regarding the impact of cultural tourism on poverty reduction	Mixed methods	Survey and interview	85 randomly selected tourism-oriented households in five villages	Economic	Moderate
African	[44]	Orock	2014	Cameroon	To determine the extent to which revenue from ecotourism is used to improve livelihoods of local community	Mixed methods	Survey and interview	200 questionnaires Interviews (number of interviews not stated) Participants not described	Economic	Poor
African	[45]	Magigi and Ramadhani	2013	Tanzania	To determine how community participated in tourism and the effects of tourism in reducing poverty	Mixed Methods	Questionnaire, interviews, observations, documentary reviews and photographs	Interview conducted with sample of villagers including residents, workers in the tourist industry and government staff	Economic	High
African	[46]	Steinicke and Neuburger	2012	Kenya	To determine the impact of alpine tourism on the economy and to assess whether income derived from community-based tourism impact on the livelihood of the community	Mixed methods	Interviews, focus group discussions and secondary data from various official sources	27 household participants interviewed from community 48 guides and porters interviewed	Economic	Moderate
African	[47]	Rotich and Obombo	2012	Kenya	To determine whether community tourism reduced poverty, identify the challenges and how local communities support and participate in tourism	Mixed methods	Survey and focus group discussions	190 respondents: 150 local residents, 20 tourists, 10 managers, 10 opinion leaders	Economic	Poor
African	[48]	Spenceley and Goodwin	2007	South Africa	To describe the economic impact of nature-based tourism on the surrounding communities	Mixed methods	Survey and one-on-one interview	1,058 respondents from 4 communities	Economic	High
African	[49]	Snyman and Spenceley	2012	Malawi Zambia and Zimbabwe	To determine the tangible benefits of tourism on local communities affected by protected area	Quantitative	Survey	Survey of staff and communities living alongside conservation areas 165 staff working in seven high-end lodges 539 community surveys in 15 rural villages including 14 different ethnic groups	Economic	High
African	[50]	Snyman and Lynne	2012	Botswana Malawi Namibia	To assess the impact of ecotourism on employment of rural communities	Quantitative	Survey	Survey of 618 community members from 25 rural communities Staff and non-staff of ecotourism camps surveyed	Economic	High
African	[51]	Snyman	2014	Botswana Malawi Namibia South Africa Zambia Zimbabwe	To examine the impact of ecotourism employment on household incomes and social welfare	Quantitative	Survey	1785 surveys in 6 countries: 385 staff interviewed in 16 ecotourism camps 1400 interviews conducted in 30 rural communities	Economic	High
African	[52]	Emptaz-Collomb	2009	Namibia	To investigate the association of tourism participation to host communities’ wellbeing and attitude towards conservation	Mixed Methods	Focus group discussions and cross-sectional survey	467 randomly selected individuals participated in the survey, stratified per conservancies, age, gender, and whether they are employed or not employed in tourism	Health and wellbeing Economic	High
African	[53]	Adetola and Adediran	2014	Nigeria	To investigate attitude of indigenous communities regarding tourism in Olumirin Waterfall	Quantitative	Cross-sectional survey and inferential statistical analysis	150 Village residents were surveyed	Economic	Poor
African	[54]	Bwalya and Kapembwa	2020	Zambia	To explore views of residents regarding the economic benefits and participation in wildlife management and conservation	Qualitative	Interviews and focus group discussions	Community interviews: 267 households from each of the three chiefdoms Key informant interviews: 3 chiefs, 2 chief representatives, 3 officials from the Zambia Wildlife Authority and representatives from conservation agencies and 3 long-term residents	Economic Ecological	Poor
African	[55]	Folarin and Adeniyi	2020	38 Sub-Saharan African countries	To examine whether tourism development reduces the level of poverty in 36 Sub-Saharan Countries	Quantitative	Econometric analysis of secondary data, three poverty indicators, Generalised Method of Moments (GMM)	Multiple economic indicators of 36 Sub-Saharan countries from 1996 to 2015	Economic	High
African	[56]	Lepp	2004	Uganda	To understand the perception of impact of tourism in the local community of Bigodi in Western Uganda	Qualitative	Interview	Interviews of 15 high ranking tourism officials and 48 residents	Economic	High
African	[57]	Meyer and Meyer	2016	South Africa	To determine the relationship between tourism and local economic development.	Quantitative	Secondary data, regression analysis	Secondary data derived from Global Insight 2015 (from 2001 to 2014) including tourism spending, Regional Gross Domestic Product (GRDP)–(representing economic development)	Economic	Poor
African	[58]	Mosetlhi	2012	Botswana	To determine the impact of the Chobe National Park on the livelihood of people and their conservation behaviours	Quantitative	Cross-sectional using systematic random sampling	Two groups of participants: 473 Household heads, 12 Key informants (representatives from community level bodies with livelihood or conservation responsibilities) and relevant local authorities	Economic Sociocultural	Moderate
African	[59]	Mutana and Mukwada	2018	South Africa	To determine whether local communities, business owners and government officials view mountain route tourism as contributing to poverty reduction	Mixed Methods	Survey and interview	Survey of 80 tourism business operators 250 interviews with local community members and local government officials	Economic	High
African	[60]	Stone	2013	Botswana	To explore the link between protected areas tourism and community livelihood; to assess changes in community needs due to participation in wildlife tourism; and to assess the relationship between tourism and community capitals	Qualitative	Semi-structured interviews	47 interviews with key government officials and randomly selected household heads	Economic Social	High
African	[61]	Lyon	2013	South Africa	To examine whether tourism contributes to sustainable development in the Waterberg Biosphere Reserve (WBR) in South Africa	Qualitative	Semi-structured interviews	35 purposely selected interviewees who were tourism influencers from the public sector, accommodation providers, business owners, civil society individuals, land claimants, representative of the hunting sector and the WBR coordinator	Economic	High
African	[62]	DeLuca	2002	Tanzania	To examine the growing role of tourism in conservation and development projects in Ngorongoro District, a part of the Serengeti ecosystem; and to analyse the role of tourism in local livelihoods and the social impacts of safari tourism	Mixed methods	Open-ended interviews, surveys, archival research, image analysis and participant observation	Social/demographic survey: 98 participants Enumerator survey: 95 Interviews: 128 (including walking tour guides, cultural Boma members, Safari camp owners, driver guides, lodge managers, etc)	Socio-economic Cultural Environmental	High
European	[63]	Polonsky et al.	2013	Turkey	To evaluate the direct and indirect impacts of business providing strategic philanthropy to heritage tourism on local communities	Mixed methods	Survey and face-to-face interviews	674 residents were surveyed 270 residents participated in face-to-face interviews	Economic Social Ecological	Moderate
European	[64]	Ciolac et al.	2019	Greece	To explore the perceptions of residents regarding the impact of mass tourism and views on mass tourism and ecotourism in three municipalities of Greece	Mixed methods	Survey and interview	Survey of 202 local residents (owners of agritourist households/guesthouses from six counties) Interviews with experts and residents	Economic Social	Moderate
European	[65]	Seraphin et al.	2018	UK	To explore the impact of events and tourism on community wellbeing	Mixed methods	Survey: Case study of a Special Interest Tourism and Event	308 online questionnaires	Economic Health and wellbeing Social	Moderate
European	[66]	Boukas and Ziakas	2016	Cyprus	To examine how tourism development and policy influence wellbeing of residents to enable inside-out approach	Qualitative	In-depth semi-structured interview Documentary analysis of official policy sources	11 interviews (purposive sample) with tourism officials, high-ranking policymakers, administrators, and tourist stakeholders	Economic Social	Moderate
European	[67]	Annes and Wright	2015	France	To explore the relationship between farm tourism and empowerment among women in France	Qualitative	Document analysis (advertising brochures and websites)	Five female participants who are current or past members of an agritourism network (sheep farming)	Sociological effects–empowerment of women and confidence Economic impact of agritourism on women	Moderate
European	[68]	Bimonte and D’Agostino	2020	Italy	To determine whether the onset of tourism directly or indirectly affected residents’ subjective wellbeing	Quantitative	Survey, Propensity Score (PS) method	Pre and peak-tourism season survey of two seaside destinations comparing wellbeing of residents (random sample from Municipal sampling register) Propensity score method	Ecological Social	Moderate
European	[69]	Chazapi and Sdrali	2006	Greece	To measure the perception of locals regarding the impact of tourism	Quantitative	Survey	350 residents in Andros 87% were permanent residents of the island with average length of stay of 25 years	Economic impact Ecological Cultural	Moderate
European	[70]	Ehinger	2016	Kyrgzstan	To evaluate the economic, social and environmental impacts of CBT on the village of Arslanbon, Kyrgyzstan	Qualitative	Interview of tourism and environmental management stakeholders and residents	43 tourism and management stakeholders 3 weeks of participant observations	Economic Ecological Social Health & wellbeing	High
European	[71]	Leu	2019	Sweden	To determine the impact of tourism on the (livelihood strategies) employment opportunities and entrepreneurship of indigenous Sami people of Sweden	Qualitative	Semi-structured interviews	13 semi-structured interviews with indigenous Sami tourist entrepreneurs	Economic impact	Moderate
European	[72]	Marković et al.	2020	Serbia Bosnia and Herzegovina	To analyse the attitudes of local communities regarding the impact of local sports event tourism	Quantitative	Survey, descriptive statistics, Chi-square test	238 respondents from the local community and sports organisers (within three municipalities)	Economic impact Social impact	Poor
European	[73]	Obradović et al.	2020	Serbia	To explore the perception and satisfaction with tourism development in the Special Nature Reserve (SNR) among local communities	Quantitative	Survey, regression analysis	152 respondents (local residents of 13 villages of the SNR)	Perceptions of sustainable development Satisfaction of local residents with tourism	High
European	[74]	Labadi	2011	UK, Poland, France	To analyse methods for the evaluation of socio-economic impacts of regeneration projects and the impacts identified; to highlight those impacts that are sound and those that do not stand critical analysis; and to make recommendations for improving impact evaluations, regeneration models and for enhancing respect for cultural diversity and social cohesion	Mixed methods	Case study (primarily secondary data)	Case 1: 107 Lowry gallery visitors, plus data obtained from existing reports on project spend, visitor numbers, and other secondary data Case 2: approx. 500 questionnaires with local population, face-to-face and written interviews landscape survey and townscape evaluation Case 3: questionnaires sent to 423 students, archival research, landscape surveys, essays written by school children, analysis of guidebooks, newspapers and promotional material Case 4: yearlong evaluation as part of the European City of Culture, analysis of ticketing and visitor data, survey on profits/turnover, face-to-face survey with 300 visitors	Socio-economic	High
Americas	[75]	McDonough	2009	Bolivia	To determine whether ethno-ecotourism is an adequate substitute economic activities for indigenous communities and how this benefits conservation.	Qualitative	Interviews, observations and secondary data	Analysis of three ecotourism projects inside or near two protected areas. Interviews of 65 residents, descriptive characteristics of residents not stated.	Economic Social	Moderate
Americas	[76]	Diedrich	2007	Belize	To explore the impact of tourism development and nature conservation among local communities	Mixed Methods	Participant observation, semi-structured interviews, secondary sources, survey	Qualitative: ethnographic observation, semi-structured interviews of key informants, secondary sources Quantitative: Random household survey of 227 residents from 5 coastal communities (sub-sample of 111 fishermen and 93 marine tour guides)	Socioeconomic Ecological	High
Americas	[77]	Spiegel et al.	2008	Cuba	To understand the impact of tourism on the local communities and examine health promotion responses	Qualitative	Focus group discussions	8 focus group discussions (4 in community being developed as a tourist destination and 4 in the established tourist community)	Health Social Ecological	Poor
Americas	[78]	Harbor and Hunt	2020	Guatemala	To examine how indigenous people negotiate tourism to generate fair and just outcomes for them	Qualitative	Ethnographic semi-structured interviews	Interviewees: 34 tourism-related business owners or employees 15 informant purposively sampled community members involved in tourism with one expatriate American citizen hotel owner.	Economic Sociocultural	High
Americas	[79]	Renkert	2019	Ecuador	To examine how community-owned tourism might benefit local community	Qualitative	Ethnographic interview	30 semi-structured interviews were conducted with Anangu community members, ecolodge staff and visitors.	Economics Sociocultural Ecological	Moderate
Americas	[80]	Stoddart, Catano and Ramos	2018	Canada	To examine the perceptions of rural communities regarding the economic, socio-cultural, and environmental impacts of tourism	Mixed methods	Survey, interviews, and field notes/observations	Telephone survey (n = 95) Informal and Semi-structured Interviews (4 key informants in Battle Harbour; 12 key informant interviews in Burin District)	Economic Sociocultural	Poor
Americas	[81]	Slinger	2002	Dominica	To determine the direct impact of ecotourism on local communities, on the diversification of the local economy and the links created by ecotourism; and protection of the environment	Mixed Methods	Survey questionnaire Semi-Structured interviews of key stakeholders	326 surveys administered to those involved in the tourism industry	Economic Ecological	Moderate
Americas	[82]	Yu, Cole and Chancellor	2018	USA	To examine the positive and negative impacts of tourism on the quality of life of local residents and its influence on residents’ support for tourism development	Quantitative	Survey, structural equation modelling (SEM)	324 randomly selected participants	Economic Sociocultural Ecological	Moderate
Americas	[83]	Ohl-Schacherer et al.	2008	Peru	To determine the economic and non-monetary impact of ecotourism on indigenous community	Mixed methods	Secondary data and informal interviews and field observation	Secondary financial data were taken from accounting records and receipts Data of handicraft sales used to estimate sales per visitors for other years. Estimates were used to calculate income required to meet needs	Economic Sociocultural	Poor
Americas	[84]	Robinson, Newman and Stead	2019	Turks and Caicos Islands	To investigate factors that influence residents’ support for tourism and identify the perceived impacts of tourism on economic, social & environmental aspects	Qualitative	Semi-structured interviews	30 household heads sampled systematically 23 direct resource users such as those working in the tourism industry and fishermen	Economic Ecological Sociocultural	Moderate
Americas	[85]	Alves et al.	2013	Brazil	To evaluate the socioeconomic impact of tourism activity focused on Dolphin provisioning	Qualitative	Interview	45 interviews 24 with residents and 21 with businessmen (Snowball sampling)	Economic Ecological	Poor
Americas	[86]	Barthel	2016	Columbia	To assess the impact of tourism on forest loss	Quantitative	Secondary data	Data derived from internet sources of data detailing forest loss to identify deforestation sites near protected areas. Data on tourist arrivals to indicate level of tourism activity.	Ecological	Moderate
Americas	[87]	Beckman and Traynor	2018	USA	To determine the economic impact of a 5-day tourist event on the host community	Quantitative	Survey, Trade Market Analysis (TMA),distance travelled method and geographic method	Surveyed (n = 833) 688 tourist groups 145 local groups	Economic	High
Americas	[88]	Cannonier and Burke	2018	Caribbean	To determine the impact of tourism on economic growth	Quantitative	Secondary data, Generalised Method of Moments (GMM) and instrumental variables method (IV)	Panel data from 15 Caribbean countries from 1980 to 2015. Tourism variables included tourist expenditures (tourist receipts), number of international tourist arrivals per capita. Six main control variables including GDP per capita, investments, trade, government consumption and inflation.	Economic	High
Americas	[89]	Lottig	2007	USA	To investigate residents’ perceptions about the environmental impact of tourism development and how these relate to perceived benefits for residents and their attitudes towards sustainable development.	Quantitative	Survey, structural equation modelling (SEM)	440 surveys were administered to Oahu residents 18 and above	Ecological	High
Americas	[90]	Oviedo-Garcia, González-Rodríguez and Vega-Vázquez	2018	Dominican Republic	To analyse the impact of tourism on poverty alleviation and inequality of income distribution of local communities	Quantitative	Secondary data, Autoregressive Distributed lag (ARDL)	Income indicators	Economic	High
Americas	[91]	Raschke	2017	Dominica Dominican Republic	To examine perceptions of the impact of whale watching on conservation and wellbeing on the human communities in the Caribbean	Qualitative	Interview	20 Participants from Dominican Republic and 11 participants from Dominica Key informants (eg. government officials)	Economic Ecological	High
Americas	[92]	Serenari et al.	2017	Chile	To explore perceptions of local people on the impact of private protected areas (PPA) and are they engaged in conservation and ecotourism.	Qualitative	Interview	85 interviews including government officials, PPA administrators and advisors, community leaders tour guides & business owners	Economic Ecological	Moderate
Americas	[93]	Aleshinloye et al.	2021	USA	To examine the influence of tourism on residents’ psychological, social and political empowerment and its impact on quality of life and place attachment	Quantitative	Survey, PLS-SEM	Systematic sampling of permanent residents living in the district of Central Orlando, Florida (survey using 5 validated scales)	Economic Sociocultural Health	High
Americas	[94]	Bennet	2009	Canada	To explore the perceptions of the Lutsel K’e Dene community, who lives in the Great Slave Lake area of Canada, regarding the benefits of tourism and the role of social economy in community development to inform establishment and planning of the park.	Qualitative	Interviews	26 interviews: Lutsel K’e members, 10 non-members and 8 external participants	Sociocultural Economic	High
South-East Asia	[95]	Utomo et al.	2020	Indonesia	To examine the relationship between rural-based tourism and local economic development	Mixed methods	Questionnaires, interview	Questionnaires to map local economic development status (number not stated) In-depth interviews with unspecified respondents	Economic Ecological Social	Poor
South-East Asia	[96]	Gunjan, Mowla and Sultanul	2020	Bangladesh	To identify community well-being as a determinant factor in sustainable tourism development; and to conceptualise and understand the link between community capitals and dimensions of social interface, and their role in contributing to community wellbeing	Qualitative	Interview	Biographical, in-depth and open-ended interviews with residents with a range of community profiles (36 interviewees) Ethnographic approach to observe religious and cultural programmes, and dependence on tourism	Social Environmental	Moderate
South-East Asia	[97]	Yergeau	2020	Nepal	To study the relationships between tourism, environmental constraints, and local monetary welfare in Nepal’s protected areas	Quantitative	Survey, multilevel modelling	1563 households in 71 wards Household surveys (with households identified via random systematic sampling)	Environmental Socioeconomic Welfare	High
South-East Asia	[98]	Chong	2020	Indonesia	To examine whether that the degree of residents’ dependence on tourism impacts perceptions and attitudes toward mass tourism	Qualitative	Case study, interview	Case study approach (4 areas) with a focus on host-perspectives (20 participants, various experiences with tourism) In-depth interviews (open ended) examining understanding, perceptions and opinion	Socioeconomic Sociocultural Environmental	Moderate
South-East Asia	[99]	Sasmitha and Marhaeni	2019	Indonesia	To analyse the influence of tourism villages development on community empowerment; to analyse the influence of tourism villages development and community empowerment on community welfare; to analyse the role of community empowerment in mediating influence of tourism development on community welfare; and to design a tourism village development strategy	Mixed methods	Observations, interview	Structured and in-depth interviews with 194 respondents	Socioeconomic Welfare	Moderate
South-East Asia	[100]	Adrija, Vicky and Pradeep	2019	India	To examine whether improved governance and appropriate investment in PAs will lead to maintaining ecological security, food security and sustainable development of society on a long-term basis	Mixed methods	Social surveys (structured interviews and questionnaires), Focus Groups (semi-structured and unstructured), Secondary data	173 (?) households from 22 villages around the tourism gates of 5 protected areas– Villages mixture of tourism and non-tourism villages (income predominantly from agriculture only)	Economics Socioeconomic Ecological	Poor
South-East Asia	[101]	Sangpikul	2017	Thailand	To examine how ecotourism tour operators and their guided tours contribute to the development of economic, social and environmental dimensions at ecotourism sites and local communities	Qualitative	Interviews and participant observations	Included two tour operators, 4–5 interviewees per operator (it is unclear why a precise number could not be provided), in roles associated with business management and tour operations 3 tours from each company were observed (6 in total)	Economic, social and environmental dimensions of ecotourism	Moderate
South-East Asia	[102]	Naidoo and Sharpley	2016	Mauritius	To consider and compare the perceptions of local people of the extent to which enclave tourism and agritourism contribute to their wellbeing	Mixed methods	Interview and survey	In-depth interviews with key informants Questionnaire-based surveys, pretested with 18 individuals 27 interviews with govt. officials, entrepreneurs, hotel managers, academic and tour operators 300 survey participants (residents)	Health and wellbeing Ecological	Moderate
South-East Asia	[103]	Sumarja, Hartoyo and Wahab	2014	Indonesia	To assess coastal area governance and its capacity to empower local communities in coastal tourism development through strengthening land rights for local communities	Qualitative	Secondary data, focus group discussions	Sample size and participant characteristics not specified	Economic	Poor
South-East Asia	[104]	Rahman	2010	Bangladesh	To evaluate the socio-economic impact of tourism development on the local community in Cox’s Bazar, Bangladesh	Qualitative	Case study, interview	Single case study Semi-structured interviews 35 respondents: entrepreneurs (10), government officials (5), local communities directly involved in tourism (10) and local communities not involved in tourism (10) (snowball sampling)	Socioeconomic	High
South-East Asia	[105]	Walpole and Goodwin	2001	Indonesia	To examine local attitudes towards protected area tourism and effects of tourism benefits on local support for Komodo National Park, Indonesia; and to assess whether receipt of tourism benefits result in more positive attitudes towards conservation	Mixed method	Case study, survey, interview	Structured questionnaire survey, randomly distributed to 401 households in two “gateway” villages Follow-up interviews	Environmental Ecological Economic Sociocultural	High
South-East Asia	[106]	Wright and Lewis	2012	Indonesia	To explore key community stakeholders’ perspectives on development, tourism, and community sustainability in Delha, Rote	Qualitative	Interview	Action Research Cycle Qualitative pilot study In-depth interviews with key stakeholders, conducted remotely (via phone or while in Australia) 11 participants (local and non-local) Purposive sampling	Social Cultural Environmental Loss of autonomy Loss of community control ‘Dynamics of exclusion’ Health and wellbeing Future sustainability	High
South-East Asia	[107]	Phelan, Ruhanen and Mair	2020	Indonesia	To examine the role of community-based ecotourism in progressing the blue economy (use of ocean resources for economic development and human wellbeing)	Qualitative	Interviews	Semi-structured interviews 15 households interviewed from three representative villages (5 from each village). 12 key informants (village heads, village committee members, government officials, chief of police, local business owners).	Economic Ecological	Moderate
South-East Asia	[108]	Ali et al.	2020	Bangladesh	To explore the impact of ecotourism on the quality of life of communities living within or near to the site	Quantitative	Survey	449 surveys were collected from purposively selected samples (respondents were engaged in tourism related occupations or businesses)	Economic	Moderate
South-East Asia	[109]	Anggraini	2015	Indonesia	To explore how local residents living in tourist areas construct a sense of place attachment and identity, how these influence attitudes and behaviours towards tourism, and its contribution to the health and wellbeing of locals	Qualitative	Focus group discussion	4 focus group interviews using different media as discussion points (25 interviewed). Digital ethnography (data from social media platforms). Photo elicitation (88 photographs from participants)	Sociocultural	High
South-East Asia	[110]	D’Mello et al.	2015	India	To explore factors that influence residents’ attitudes towards tourism development	Quantitative	Survey	809 survey questionnaires of residents in Goa 18 years and above	Sociocultural	High
South-East Asia	[111]	Vajrakachorn	2011	Thailand	Relevant to this study, the aim was to identify the success factors of a CBT destination from the viewpoint of the local community	Mixed methods	Survey, interviews, and participant observation	Survey: 193 respondents 32 key informants were interviewed (85% government officials)	Economic Sociocultural	High
Western Pacific	[112]	Ma and Wen	2019	China	To understand community preferences for different conservation and development policies from the perspective of local households; and to evaluate participation willingness and stated preferences regarding the establishment of national parks (NPs), ecotourism development, ecological public welfare forest compensation, and provision of ecological jobs.	Mixed methods	Interview, survey	Preliminary interviews (communities, experts, and managers) and consultation with experts and NP Managers to inform questionnaire design, which was administered face-to-face with randomly selected households (219 valid questionnaire surveys were collected in four counties near or within four Nature Reserves)	Socio-economic Poverty reduction and increased welfare Environmental	High
Western Pacific	[113]	Kry et al.	2020	Cambodia	To assess local livelihood assets in Kampong Phluk Community before and after the introduction of the community-based ecotourism governance system; and to assess the relationship between ecotourism development governance systems and local livelihoods in the Kampong Phluk community located in the Tonle Sap Great Lake.	Mixed method	Survey, interview	75 household surveys from local households in Kampong Phluk (conducted based on village and main occupation) Interviews with government officials, the Head of the Kompong Khluk commune, village Heads, the boat community leader, and the fishery community (number of interviews unclear)	The study was designed around the SD framework and five types of capital: natural, physical, human, financial, and social	High
Western Pacific	[114]	Bakri and Jaafar	2015	Malaysia	To revisit how tourism development affects residents’ quality of life and to review the willingness of society to accept tourism development occurring in their area	Quantitative	Survey	398 self-administered questionnaires Participants aged 18 and over and working in various fields on Langkawi Island	Quality of life, based on emotional wellbeing, community wellbeing, H&S wellbeing, material wellbeing and cost of living	Poor
Western Pacific	[115]	Catibog-Sinha	2013	Philippines	To examine the issues and challenges of promoting sustainable island tourism in Puerto Galera	Qualitative	Field observations, semi-structured interview, and secondary data review	Semi-structured interviews with key govt. officials and park managers at local and national level (no precise number provided)	Environmental impacts of tourism Enhancement of biodiversity and cultural heritage	Poor
Western Pacific	[116]	Kuo and Chiu	2006	Taiwan	To develop a new assessment approach based on SEA in combination with HIA and apply it to agritourism policy in Taiwan; and to identify the potential impact on people’s health as well as environ- mental impacts of a policy	Quantitative	Survey	The Delphi-Indicator Approach (5-stage): Questionnaire design, questionnaire administration, estimation of impact assessment system, impact assessment and summary. Stage 2 (interviews) involved 18 experts, including discipline professors, leaders of NGOs and officials Stage 3 revolved around a workshop composed of 17 experts.	Health and Wellbeing Environmental impacts	Poor
Western Pacific	[117]	Dyer, Aberdeen and Schuler	2003	Australia	To explore the impacts of tourism on an Australian Indigenous community, the Djabugay people, whose traditional land is located near Cairns, far north Queensland.	Qualitative	Participant observations, open ended interviews, and document analysis	Structured open-ended interviews with 49 park employees (non-management) and 7 managers Informal interviews with the Djabugay community	Political Economic Social	High
Western Pacific	[118]	Suntikul	2011	Laos	To explore the effects that contact with tourists have on the values and practices of the monks of Luang Prabang and, in turn, how these changes have affected the spirit of the place	Qualitative	Observation, survey, interview	On-site observations, surveys (with monks and tourists) and interviews (with religious leaders) Surveys with 152 monks Surveys with 54 tourists–no characteristics provided Interviews–unspecified	Cultural sustainability	Moderate
Western Pacific	[119]	Suntikul	2008	Laos	To examine the impacts of heritage preservation policy and practice on businesses in the UNESCO listed town centre of Luang Prabang	Qualitative	Interview	29 semi-structured interviews, 13 with Laotian owners of tourism-related businesses and 9 with foreign owners of tourism-related businesses	Cultural sustainability Sustainability of Built Heritage Economic growth	Moderate
Western Pacific	[120]	Ahmad and Ma	2021	Hong Kong, Singapore, and South Korea (Taiwan was omitted due to a lack of data)	To explore the role of tourism development in pollution emissions by investigating two influencing mechanisms—the industry substitution effect and energy substitution effect—in the context of Asian Tigers	Quantitative	Survey, econometric modelling	Econometric modelling Data Source: annual data collected from World Development Indicators and the World Bank	Environmental sustainability and pollution reduction/emissions	High
Western Pacific	[121]	Deng, Liu and Hu	2021	China	To explore the effects of tourism development on shrinking cities	Quantitative	Survey, Generalised method of moments (GMM)	Panel data of 54 shrinking cities 2-step dynamic panel estimation using system-GMM 2-stage least square (2SLS) estimators	Regional economic growth Revival of shrinking cities Socio-economic prosperity	Moderate
Western Pacific	[122]	Falatoonitoosi, Schaffer and Kerr	2021	Australia	To determine perceptions of stakeholders regarding the role of sustainable tourism development in enhancing prosperity of destination community	Mixed Methods	Interview, survey	Four-stage convergent interviews of 20 experts from tourism and social science sectors. Survey of 171 stakeholders from 5 categories (communities, public sector, private sector, NGOs environmental and conservation groups, tourism organisations)	Prosperity with the following components: Quality of life Sociocultural empowerment Environmental quality Economic growth Tourist satisfaction Attractiveness of destination	High
Western Pacific	[123]	Liang, Umezaki and Ohtsuka	2003	China	To elucidate the changing living conditions and environment uses of the people in a Li-speaking village situated in the centre of tourism development.	Qualitative	Observations, interview	Insufficient detail provided regarding sample size, participants and/or their characteristics	Positive: • Poverty alleviation • Health and Wellbeing Negative: • Environmental degradation • increasing socio-economic differentiation	Poor
Western Pacific	[124]	Liu et al.	2012	China	To investigate the diverse benefits that households receive from the development of nature-based tourism	Quantitative	Survey	Empirical household economic model based on questionnaire surveys and personal interviews Representative sample of 220 local households Tourism households were identified if at least one member was working directly with the tourism sector	Socioeconomic impacts of tourism and their distributional patterns. Household livelihood asset portfolios consisted of financial capital, human capital, natural capital, physical capital	High
Western Pacific	[125]	Moscardo et al.	2013	Australia	To investigate the tourism impacts on community wellbeing in three Australian destinations; and to assess the implications of their findings for sustainable tourism planners and researchers, and especially for resident perceptions research	Qualitative	Interview	Semi-structured interviews with destination community stakeholders in three regional locations (interviews with 25 key informants from three regional communities) All informants were outside of tourism (i.e. health, local govt., finance, arts/cultural)	Wellbeing, organised around 7 types of capital (financial, natural, built, social, cultural, human, and political)	Moderate
Western Pacific	[126]	Mules	2005	Australia	To assess the extent to which tourism to Kosciuszko NP has economic impacts on neighbouring regions as a group	Quantitative	Survey, Geographic Allocation of Tourism Expenditure (GATE) model	Economic modelling using self-administered surveys completed by visitors over a 12-month period (3096 surveys returned)	Economic impacts for regional economies and for gateway communities	Moderate
Western Pacific	[127]	Murray	2017	Japan	To consider how new subjectivities are produced when host communities see themselves through the lens of visiting tourists; and to explore how Okinawans’ sense of place and identity are transformed as their language, landscapes, and wildlife are reconstituted as cherishable yet vulnerable resources.	Qualitative	Observation, interview	Ethnographic fieldwork or ‘landscape ethnography’- Participant observation; Formal and informal semi-structured interviews were undertaken with govt. officials, academics, non-profit directors and affiliates, tourists, guides and museum employees.	Socioeconomic Environmental impacts Cultural revival	High
Western Pacific	[128]	Qiu et al.	2019	Hong Kong	To develop and validate a framework for assessing economic sustainability from the perspective of local stakeholders	Mixed Methods—multi-step process	(1) literature search (2) in-depth interviews (3) panel of experts (4) pilot (5) telephone surveys (6) analysis	Interviews: 12 major stakeholders in the Hong Kong tourism industry Panel: five scholars with expertise in sustainability Pilot: 80 Hong Kong University students Surveys: 1938 Hong Kong citizens (18 years or over)	Economic Sustainability	Poor
Western Pacific	[129]	Shahbaz et al.	2018	Malaysia	To explore the relationship between tourism development and financial development by incorporating economic growth and effective exchange rate as additional determinants in finance demand function of Malaysian economy	Quantitative	Survey, econometric modelling	Econometric modelling Data drawn from the World Development Indicators on Malaysian economy. Data on tourist arrivals, receipts and expenditure is drawn from Tourism Statistics, Ministry of Tourism Malaysia.	Economic Growth and Financial Development	High
Western Pacific	[130]	Shi et al.	2019	China	To provide insights into the importance of interregional tourism policies and strategies for inbound tourism development in China	Quantitative	Survey, Moran’s I model	Balanced data panel of 31 Chinese provinces. Spatial econometrics using Moran’s *I* model to determine urban-rural income disparity. Data drawn from China Statistical Yearbook and supplemented with Wind Information data.	Economic Equity	Moderate
Western Pacific	[131]	Su, Wall and Xu	2016	China	To determine the extent to which tourism strategies are contributing to local livelihoods in three rural villages at Mount San- qingshan World Heritage Site, China	Qualitative	Interview, focus group discussion, observation, secondary data	Semi-structured interviews and face-to-face focus group discussions with residents and leaders in 3 sub-villages, plus participant observations. Interviews: 2 x key management officials, 1x community leader 22 x village residents 1 x focus group with Yingiang major and the 4 members of the village committee Site development plans and policy documents also reviewed and analysed.	Community livelihoods and wellbeing Balancing heritage conservation with community needs Sustainable livelihood outcomes	Poor
Western Pacific	[132]	Yang and Chen	2006	Taiwan	To examine nature-based tourism (NBT) impacts from business managers’ perceptions in economic, cultural and environmental aspects To understand the relationship among socio-demographic characteristics, type and level of involvement and participants’ perception of impacts	Quantitative	Social survey, utilising a multi-phased approach: (1) individual interviews to identify the role of nature-based tourism in I-Lan (2) a short survey (3) larger on-site survey (4) follow-up interviews	Interviews: number unknown Survey pre-tested with 60 college students and 14 business managers. On-site survey: 316 surveys collected (286 deemed usable). Participants from 15 business types (hotels, restaurants, leisure farms and gift shops, etc.) and distributed across 12 administrative districts.	Economic Environmental Cultural	High
Western Pacific	[133]	Zhang et al.	2020	China	To develop and test a multidimensional scale to evaluate the perceived social impacts from tourism on social capital from sustainable community-based tourism in China	Quantitative	Multi-phased approach based around a survey questionnaire: (1) literature review (2) pilot questionnaire (3) Panel assessment (4) 2nd pilot study (5) resident survey (6) PCA and varimax rotation	Pilot questionnaire with 60 respondents Panel assessment with 7 members (academics, govt. official, tourism operators and village committee members) Second pilot with 60 residents Survey administered to those 18+ and permanent residents of Fanhe village working in the tourism industry. 500 surveys were administered; 430 were deemed usable.	Social capital and sustainable tourism: • community efficacy • Community belonging • Traditional social regulation • community cohesion • social networks • community competence	High
Western Pacific	[134]	Zhao	2020	China	To explore the tourism–poverty nexus; and to ascertain the moderating role of institutional quality in understanding this nexus	Quantitative	Survey	Comparative analysis using a system generalised method of moments technique Dynamic panel data modelling Panel data for 29 Chinese provinces Data obtained from statistical yearbooks and research institute reports	Poverty alleviation	High
Western Pacific	[135]	Zhao and Xia	2019	China	To examine whether tourism affects poverty reduction based on the panel data of Chinese provinces for the period from 1999 to 2014	Quantitative	Survey, econometric modelling	Econometric modelling of 29 provinces. Tourism, income, and rural development data all obtained from statistical yearbooks.	Poverty reduction	High
Western Pacific	[136]	Zuo and Huang	2020	China	To provide empirical evidence of how tourism triggers economic growth from a meso-level structural perspective.	Quantitative	Survey, Shift-Share Analysis (SSA)	A modified shift-share analysis (SSA) using the Lewis Hypothesis. Examined overall economy and four main sectors: agriculture, manufacture, the service sector (excluding tourism) and the tourism sector. Data sources include survey reports and statistical yearbooks.	Economic Growth	High
Eastern Mediterranean	[137]	Refaat and Mohamed	2010	Egypt	To explore the status of pottery handicraft in Tunis village and assess its role both as a tool for tourism promotion and for achieving sustainable tourism development in the village	Mixed methods	Survey, interview, secondary data	Survey 1 administered to 40 crafters (local residents) Survey 2 face-to-face interview with 15 tourists Survey 3 administered to 30 non-crafters (local residents) Secondary data included published material and unspecified internet sources	Socio-economic Environmental	Poor
Multiple	[138]	Enilov and Wang	2021	Multiple countries	To investigate the relationship between monthly Tourist Arrivals (TA) and quarterly real GDP growth (Economic Growth—EG) by using the MF-VAR approach proposed by Ghysels et al. (2016); and to provide new global evidence for the causal relationship between international tourist arrivals (TA) and economic growth (EG).	Quantitative	Survey, MF-VAR method	Econometric methods, using the MF-VAR approach developed by Ghysels et al. (2016). Annual log differenced data of monthly international TA and quarterly real GDP per capita Data set: 23 countries, including 9 of the 10 most popular destinations.	Economic	High
Multiple	[139]	Nguyen et al.	2020	Multiple countries	To investigate the potential effect on income inequality by using the GINI index after tax and transfer to examine the influence of domestic and international tourism on inequality in a global sample of 97 countries between 2002 and 2014.	Quantitative	Survey, econometric modelling	Econometric techniques Panel data from 97 countries over the period 2002–2014 For proxy of income inequality, the GINI indices for after tax and transfer used World Development Indicators World Governance Indicators World Travel and Tourism Council database	Economic	High
Multiple	[140]	Qureshi et al.	2017	Multiple countries	To examine the relationship between sustainable tourism, energy, health, and wealth in a panel of 37 tourists’ induced countries that covered around top 80 international tourist destination cities.	Quantitative	Survey, econometric modelling	Econometric modelling Data from World Development Indicators published by the Word Bank for health expenditure, improved sanitation facilities, energy use, GDP/capita, foreign direct investment net inflows, international TAs, international TDs, international tourist receipts/expenditure and total greenhouse gas emissions (1995–2015 for 37 countries)	Eco-environmental	High
Multiple	[141]	Thinley	2010	Bhutan Canada	To investigate factors that hinder community participation in park management and development in Jigne Singye Wangchuck National Park, Bhutan; and to explore the experiences and perceptions of the community of Nain, Canada on the impact of participation in park management	Qualitative	Interview Secondary data	Review of tourism and conservation policies 20 participants were interviewed (11 were employed by the government or national park)	Economic Sociocultural Ecological	High

#### Translation and synthesis of studies

**Fig 2:**
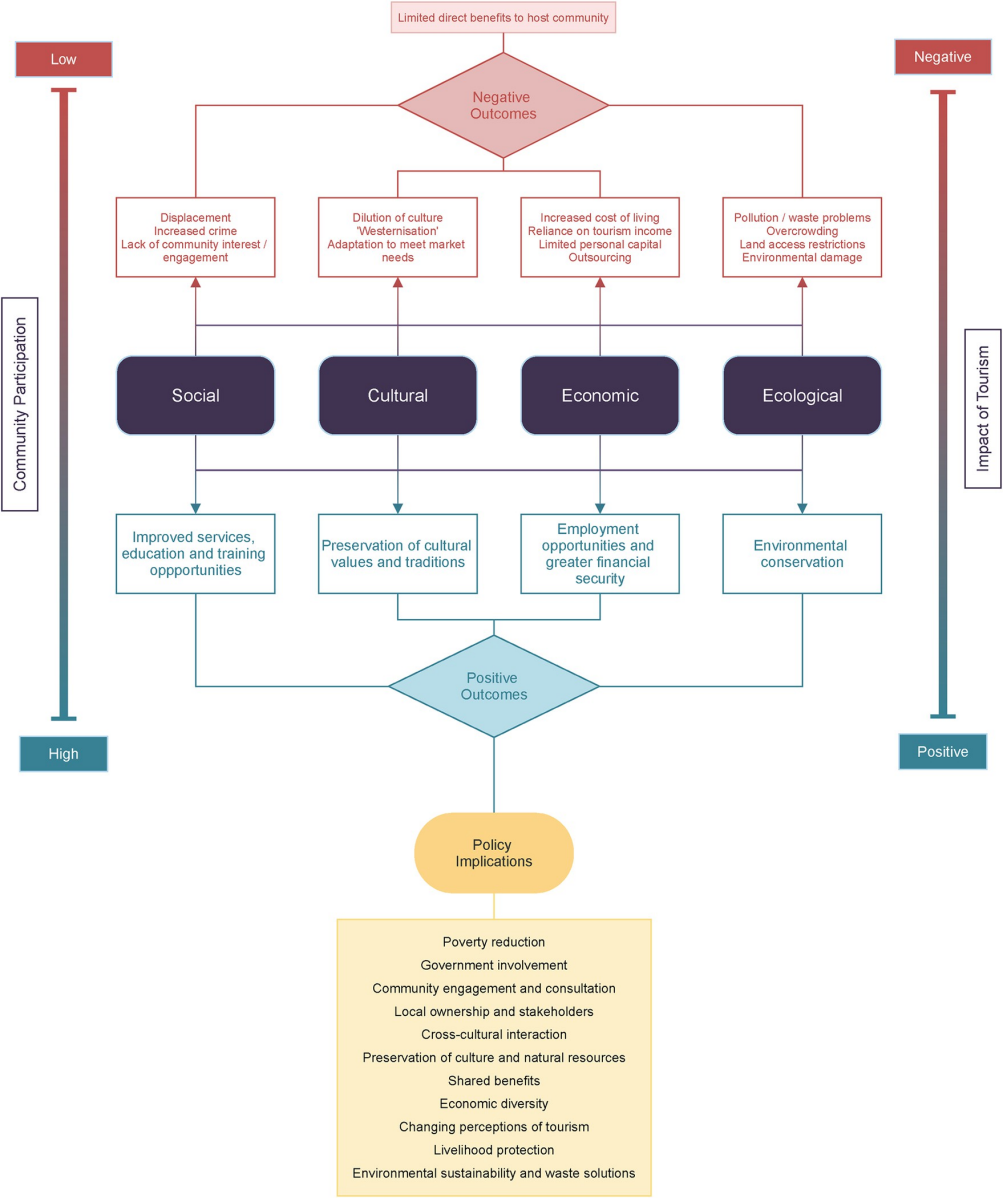
Theoretical framework and conceptual model of emerging research themes.

Themes and, where relevant, sub-themes within each study were considered and compared to the next study in a process repeated for all included studies. Such translation of studies compares and matches themes across a corpus of material, and usually involves one or more of three main types of synthesis: reciprocal translation, refutational translation, and line of argument [37]. Themes were condensed and streamlined into main thematic areas, in addition to outlining common topics within those thematic areas. The primary researcher (CB) undertook this process with discussion, validation and confirmation of themes and topics from three other researchers (EW, HS and AR). Translation between studies and the resulting synthesis of research findings followed the process of the emergence of new interpretations and conceptualisation of research themes. A line of argument was also developed, and a conceptual model produced to describe the research findings, which is shown in Fig 2. Both the line of argument and conceptual model were agreed upon by all authors.

## Results

A total of 102 studies were included in the analysis. Of these, 25 studies were conducted in the Western Pacific region, 23 in the African region, 20 in the Region of the Americas, 17 in the South-East Asia region, 12 in the European region, and 1 in the Eastern Mediterranean region. The remaining 4 studies reported on multiple regions. This may at first seem surprising given the prominence of European cultural heritage on registers such as the World Heritage List, which includes 469 cultural sites located Europe (equivalent to 47.19% of all World Heritage Properties that are recognised for their cultural values). However, any studies focusing on Europe that did not also examine sustainable community development and the overall health and wellbeing of local host communities were screened out of this systematic review in accordance with the abovementioned inclusion and exclusion criteria. Results of the data extraction and quality assessment across all included studies are presented in Table 1. Of the included studies, 24 used a mixed methods design, 22 studies were qualitative, 36 were quantitative and 20 were grey literature (see Table 1 for more detail regarding the type of methods employed). Of these, 48 studies were assessed as high quality (>75), 32 as moderate quality (50–74) and 22 as poor quality (<50).

**Fig 3:**
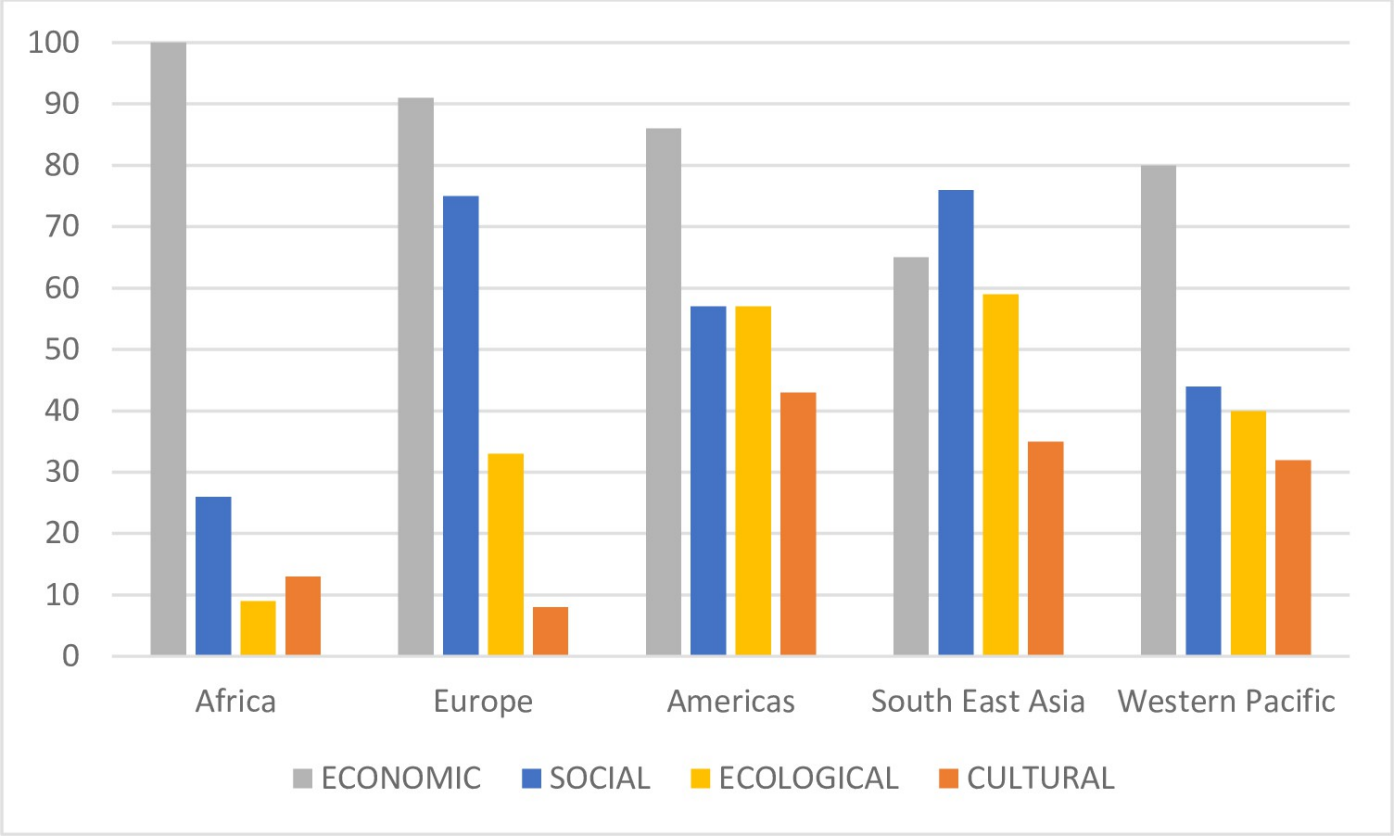
The proportion of studies that investigated the four main determinants of health and wellbeing (social, cultural, economic and ecological) in each region.

The major health and wellbeing determinant themes emerging from the included studies were grouped according to social, cultural, economic, and ecological health determinants. Fig 3 presents the proportion of included studies that investigated each of the four health determinants when assessed by WHO region. A large proportion of economic studies was shown across all regions, although this focus was surpassed by the social health determinant in the South-East Asia region (Fig 3). Studies on the social health determinant also yielded a strong proportion of studies across most other regions, although notably not in the African region. This was closely followed by an ecological focus among the Americas, South-East Asia and Western Pacific regions. The Americas had the highest proportion of cultural studies, with the European region being the lowest proportionally (Fig 3).

More specifically, for studies focused on Africa, 100% of the publications included in this review explicitly investigated the economic benefits of tourism on wellbeing (74% of them exclusively), with European-focused studies reflecting a similarly high interest in economic wellbeing (91% of publications). Across the Americas, economic determinants of wellbeing were investigated in 86% of publications and in the Western Pacific, methods to investigate this variable were built into 80% of included studies. By comparison, this research demonstrates that only just over two thirds of articles reporting on the South-East Asia region shared this focus on economic determinants (65% of publications). Instead, social determinants of wellbeing form a stronger component of the research agenda in this region, with 76% of publications investigating this theme in studies that also tended to consider multiple drivers of health. For example, in 47% of publications reporting on the South-East Asia context, at least three themes were integrated into each study, with particular synergies emerging between social, economic and ecological drivers of wellbeing and their complex relationships.

Similarly, 47% of publication reporting on the Americas also included at least three health determinants. Research outputs from these two regions demonstrated the most consistently holistic approach to understanding wellbeing compared to other regions. In Africa, only 13% of the papers reviewed incorporated three or more themes; in the Western Pacific, this figure is 32% and in Europe only 8% of research outputs attempted to incorporate three or more themes. It seems unlikely that the multidimensional relationship between socio-economic and ecological sustainability that is always in tension could be adequately explored given the trend towards one-dimensional research in Africa, the Western Pacific and particularly Europe.

The associated positive and negative impacts of heritage tourism on each of the health and wellbeing determinants are then presented in Table 2, along with the considered policy implications. Some of the identified positive impacts included improved access to education and social services, greater opportunities for skill development and employment prospects, preservation of culture and traditions, increased community livelihood and greater awareness of environmental conservation efforts. Negative impacts of tourism on host communities included forced displacement from homes, environmental degradation and over-usage of natural resources, barriers to tourism employment and reliance on tourism industry for income generation and economic stability, dilution and loss of cultural values and practices, civil unrest and loss of social stability, increased rates of crime and disease and lack of direct benefit to local communities. Both positive and negative impacts across each health and wellbeing determinant had acknowledged implications on policy development, many of which revolved around governance and ownership of tourist activities, participation of the local community in tourism sectors and active management of environmental protection programs. Such themes are shown in Table 2.

**Table 2:** Major themes identified from translation and synthesis of included studies.

Health determinant	Positive impact	Negative impact	Policy implications
Social	• Contributions to community welfare through improved health and social services • Social mobility • Better access to education • Perceived involvement in tourist activity implementation • Enjoyment of tourism benefits • Balanced and fair distribution of benefits • Training and upskilling opportunities • Increased interaction with outside world • Tourism development correlated with reduced poverty • Empowerment of women in agritourism • Improved cross-border relationships and cooperation • Provided incentive for residents to return to local areas • Development of more effective communication and negotiation skills through coordination and management of tourism lodges	• Increased crime/unlawful activities, prostitution, corruption, and rates of divorce • Racism affecting quality of life • Displacement of community and/or separation of family structure • Restrictions due to heritage sites resulting in threat and damage to local residents from wildlife • Benefits not shared with local communities • Increased outmigration of young people due to poverty and lack of livelihood • Increased demand for housing • Civil unrest and lack of government support • Lack of direct benefits to community • Increased disease transmission, introduction of exotic diseases and competition for resources • Increased disruption and conflict among local communities due to tourist presence • Changing values due to economic and social disparities between community members and tourists • Increased stress, alcoholism, tobacco use, drug addiction and obesity	• Tourism significantly reduces poverty and may be used as a poverty-reducing strategy in poverty-stricken countries • Participating in park governance and having perceived control and access to park resources are predictors of livelihood effects • Typically, locals are workers and owners are foreigners • While theoretically local residents were acknowledged as important factors in tourism, there was no specific policy or effort from stakeholders to address their needs and concerns • Development of programs with community involvement to address issues which increased the capacity of communities in decision-making and planning • Increased development of resources and programs for communities to address social and health issues • Communal governance of ecotourism allowed women to have equal employment opportunities traditionally held by men • Some members of the community were disempowered by lack of participation in decision-making • Greater welfare corresponds to those self-employed in the tourism industry
Cultural	• Preservation, rejuvenation and celebration of culture and/or cultural practices • Preservation of rural social structures, ways of life, customs and traditions • Enhanced market linkages, diversification and opportunities • Exchange of information between tourists and locals reduced isolation and reserved culture • Improved restoration of cultural and heritage structures • Opportunity to showcase culture • Tourism entrepreneurship offered a way to foster place attachment and feelings of belonging to the Indigenous homeland • Promotion of destination identity • Renewed interest in cultural history, identity and pride of village • Erosion of cultural heritage slowed because of tourism	• Erosion of cultural norms and values • Minimal input in decision-making • Threat of relocation away from ancestral land • Purchasing of locally produced goods low • Conservation restriction from the ‘right to hunt’ • Cultural practices may be deemed incompatible with conservation practices • Tourism blurred traditional symbols of cultural heritage (e.g. traditional fabric colours) as they cater to market demands of tourists • Traditional language use marginalised • Rise of ethnoreligious conflict • Local concern over gradual dilution of cultural practices and tourist needs prioritised over host needs • Loss of cultural authenticity and increasingly Westernised heritage experience	• Sensitivity to local cultural traditions and beliefs• Cultural issues not always addressed in tourism development decisions • Branding and symbolic transformation of the historic urban area consistently important in regeneration strategies • Culture may be taken for granted in driving economic growth, leading to optimism bias in regeneration projects• Once obtained, foreign investors are not obligated to maintain traditional cultural rights or practices associated with destinations• Consideration of cross-cultural interaction• Lack of current influence in management decisions of Indigenous communities due to minority shareholding, minimal voting power and lack of representation in management positions
Economic	• Employment opportunities and provision of income • Increased diversity and reliability of household income for food security and housing • Development of infrastructure, including transport • Increased profits for local tour operators • Housing affordability • Increased community livelihood • Greater income from tourism compared to traditional farming and fishing • Gateway for more lucrative foreign jobs and training • Increased number of people above the poverty line • Increased trade and investment • Maintenance of capital and/or locally reduced capital leakage • Combination of agricultural and tourism activities increasing profitability • Ecotourism provided positive linkage with other sectors (e.g. agriculture, forestry, fishing, manufacturing) which strengthened economic growth • Foreign exchange and increased taxable income for the community • Increased land value • Revival of shrinking cities through economic stimulation • Statistically significant positive effect on poverty reduction and alleviation • Important role in economic growth of developing countries	• Reliance on tourism for income/economic stability of a region or country • Lack of financial and personal capital to engage in tourism-related activities • Limited livelihood improvement from ecotourism-generated income • Low revenue from ecotourism • Continued poverty of local community • Barriers to poverty alleviation, including lack of education, required language skills and experience of tourist operators • No guarantee of jobs being good quality, well-paid or sound working conditions • Lack of skilled labour may restrict positive economic impact • Low to no income diversification during low tourism seasons • Widened income inequity among community members • Persistent cost due to proximity of protected wildlife • Increased land prices • Increased prices of essential goods and services • Difficulties of local residents to enter tourism industry • Big business may benefit from tourism expenditure, but local residents may suffer	• With increasing economic benefits, negative attitudes of communities changed • Many businesses owned and controlled by privileged few • Limited government involvement in the formation of linkages between tourism and other sectors to strengthen the economy • Government infrastructure currently inadequate to meet needs of mass tourism / tourist hotspots • Recommended that locals able to participate in tourism policy formation
Ecological	• Employment in ecotourism improved attitude towards conservation • Agritourism promoted conservation of nature • Increased awareness and education about environmental conservation of natural resources and forest health resulting from knowledge exchange with tourists • Attractiveness of the area improved • Decreased deforestation • Revenues can be fed back into conservation and management of protected areas • Tourism industry can reduce long-term carbon emissions	• Forced displacement due to land seizure/protection • Limited access to cattle grazing areas • Wildlife crop raiding and attacks • Pollution (lack of sufficient waste disposal) • Overcrowding and traffic congestion • Low level of participation in conservation • Environmental concerns largely ignored in favour of economic gains • Excessive resource usage e.g. water overconsumption • Environmental damage in high-traffic tourist areas • Environmental impact consideration often neglected in tourism development • Residents concerned about environmental impact such as plastic waste and waste disposal • Landscape problems e.g. landslides, mudflows, and overall environmental quality concerns	• Limited community consultation and participation in the management of protected areas and policies governing them • Community tourism governance helped promote stewardship of environmental resources • Support for tourism was greater amongst those involved in planning and decisions and there was perceived improvement in community relations; while support was lower in those who perceived negative environmental impact. • Governments more invested in large-scale tourism compared to small-scale tourism • Management of protected ecosystems should ensure sustainability of the ecosystem • Government support for ecotourism improved community participation and quality of life • Recommended that policy be developed to address sustainable use of the natural environment in tourism-related practices

Recent thematic trends can be observed in Table 3, whereby the percentage of research outputs that investigate economic drivers of health and wellbeing produced since 2019 are shown. In Africa, Europe and the Americas, the proportion of outputs investigating economic health determinants since 2019 is the smallest (Table 3), being 17% in Africa and the Americas, and 36% in Europe, respectively. On the contrary, 50% of Western Pacific region studies since 2019 had research focused on the economic drivers of wellbeing in relation to heritage tourism. Moreover, 65% of studies included economy-focused research in South-East Asia, with more than half of those outputs produced in the last two years (Table 3).

**Table 3:** Research outputs investigating economic drivers of health and wellbeing since 2019.

Region	% of ECONOMY theme produced since 2019	Overall % of papers investigating ECONOMIC health determinants
Africa	17	100
Europe	36	91
Americas	17	86
South-East Asia	55	65
Western Pacific	50	80

The proportion of research outputs where local community members were asked to give their opinions as participants is presented in Table 4, where they were invited to co-lead the research but were excluded from data production. In the Western Pacific region, there was a relative lack of participation (either as researchers or stakeholders) by local communities in the studies included in this review. Meaningful modes of community participation in the South-East Asian region can be calculated to 65%, more closely in line with Africa, Europe and the Americas (Table 4).

**Table 4:** Proportion of research studies with participation from host communities.

Region	*With* community participation (%)	*Without* community participation (%)	Community participation *unclear* (%)	*Designed with* community participants (%)
Africa	70	22	8	0
Europe	83	17	0	0
Americas	67	24	9	0
South-East Asia	59	29	6	6
Western Pacific	47	52	1	0

## Discussion

This systematic review is the first of its kind to explicitly consider the relationships between heritage tourism and host communities; specifically, the impact of tourism on host communities’ capacity to flourish and support long-term health and wellbeing. Such impacts were found to be both positive and negative, with either direct or indirect consequences on the development of local governance policies. Our synthesis revealed that there are important regional variations in the way that determinants of health–social, cultural, economic or ecological–drive tourism research agendas. They commonly included considerations of social dynamics, access and health of the local community, empowerment and participation of host communities in tourism-based activities and governance, employment opportunities, preservation or erosion of culture, and environmental influences due to tourism promotion or activity.

Economic impacts represented the strongest focus of the studies include in this review, often to the detriment of other cultural or environmental considerations. With the exception of South-East Asia, studies focused on all other WHO regions (Africa, Europe, the Americas and the Western Pacific) were overwhelmingly built around attempts to understand economic variables as determinants of health and wellbeing, and in some instances were likely to focus on economic variables in lieu of any other theme. Given the steady growth of an interest in economic variables in South-East Asia since 2019, it is plausible that this will soon represent the largest concentration of studies in that region, too.

This trend towards emphasis on economic influences is problematic given that some of the emerging impacts from tourism-related practices identified in this review were found to be common across multiple determinants of health and thus not limited to economic health alone. For example, the limitation placed on access to prime grazing land for cattle belonging to local residents was perceived to be a negative impact both ecologically and economically [60, 141]. This may be considered detrimental from an environmental standpoint due to the alteration of the local ecosystem and destruction of natural resources and wildlife habitat, such as the building of infrastructure to support the development of tourist accommodation, transport, and experiences.

Economically, the loss of grazing land results in reduced food sources for cattle and consequently a potential reliance on alternative food sources (which may or may not be accessible or affordable), or in the worst-case scenario death of cattle [92]. In turn, this loss of cattle has an adverse impact on the financial livelihood of host communities, who may rely on their cattle as a sole or combined source of income. Considered in isolation or combination, this single negative impact of tourism–reduced grazing access–has flow-on effects to multiple health determinants. Therefore, it is important to consider the possible multifactorial impacts of tourism, heritage or otherwise, on the host communities involved (or at least affected) given they may have a profound and lasting impact, whether favourable or not.

The potential interrelationships and multifactorial nature of heritage tourism on the health and wellbeing of host communities were also identified among a number of other studies included in this review. For example, a study from the Western Pacific Region explored connections between the analysis of tourism impacts, wellbeing of the host community and the ‘mobilities’ approach, acknowledging the three areas were different in essence but converging areas in relation to tourism sustainability [125]. That said, the cross-over between social determinants was not always observed or presented as many studies primarily focused on a single health domain [43–51, 53, 55–57, 59, 61, 71, 74, 86–90, 103, 104, 108–110, 118, 130, 134–136, 138–140]. Some studies, for instance, focused on poverty reduction and/or alleviation [134, 135], while others focused solely on cultural sustainability or sociocultural factors [109, 110, 118], and others delved only into the ecological or environmental impacts of tourism [86, 89]. As noted above, the majority of studies that focused on a single health determinant considered economic factors.

A common theme that spanned multiple health domains was the threat of relocation. Here, local communities represented in the reviewed studies were often at risk of being forced to relocate from their ancestral lands for tourism and/or nature conservation purposes [41, 60, 80, 131]. This risk not only threatens their way of life and livelihood from an economic perspective, but will also have social implications, jeopardising the sustainability and longevity of their cultural traditions and practices on the land to which they belong [41, 60, 80, 131]. Moreover, it may have ongoing implications for the displacement of family structures and segregation of local communities.

Importantly, this systematic review revealed that cultural determinants of health and wellbeing were the least explored in every region and were in many instances entirely omitted. This is at odds with the increasingly prevalent advice found in wider heritage and tourism academic debates, where it is argued that cultural institutions such as museums and their objects, for example, may contribute to health and wellbeing in the following ways: promoting relaxation; providing interventions that affect positive changes in physiology and/or emotions; supporting introspection; encouraging public health advocacy; and enhancing healthcare environments [142–144]. Likewise, Riordan and Schofield have considered the cultural significance of traditional medicine, citing its profound importance to the health and wellbeing of the communities who practice it as well as positioning it as a core element of both local and national economies [145].

Of greater concern is the finding of this review that of the relatively small number of papers investigating cultural health determinants, many recorded profoundly negative and traumatising outcomes of tourism development, such as a rise of ethnoreligious conflict, loss of ancestral land, a dilution of cultural practices to meet tourist demands, and a loss of cultural authenticity [41]. Consequently, comparative studies that focus on cultural determinants, in addition to economic and environmental determinants, are currently lacking and should therefore be prioritised in future research. In fact, only one fifth of those papers included in this review adopted the qualitative approach needed to probe the socio-cultural dimensions of health. Novel qualitative research methods to investigate community health are therefore a major research lacuna.

Just as solely equating community health and wellbeing with economic flourishing is problematic, so too is assuming that health is reducible only to clinical care and disease [146], given that "[i]deas about health … are cultural” [146]. Early indications of an acceptance that culture and heritage might be central to community health and wellbeing can be found in UNESCO’s 1995 report, *Our Creative Diversity*: *Report of the World Commission on Culture and Development* [147]. More recently, this notion is evidenced in the 2019 *Operational Guidelines for the Implementation of the World Heritage Convention* [148] and the 2020 *Operational Directives for UNESCO’s Convention for the Safeguarding of Intangible Cultural Heritage* [149], both of which indicate the need for a major shift in research foci towards cultural determinants of health and wellbeing if research is to keep pace with assumptions now operating within international policy [148, 149].

Although Africa, Europe and the Americas are the three regions with the highest proportion of papers investigating the economic benefits of tourism on health and wellbeing, these regions are also the most responsive to the above recommended changes in policy and debate (see Table 3). In these three regions, the proportion of outputs investigating economic health determinants since 2019 is the smallest, demonstrating a recent decline in research that is persuaded by the *a priori* assumption that economic wellbeing automatically equates to cultural wellbeing. Despite demonstrating the most holistic approach to understanding health and wellbeing across all the themes, an upwards trend in economy-focused research was identified in South-East Asia, since more than half of the economic outputs were produced in the last two years. Such a trend is potentially problematic for this region because it may reinforce the notion that the main benefits of tourism are direct and financial, rather than refocusing on the tension created by indirect effects of tourism on quality of life and community wellbeing.

Conversely, this review demonstrates that the Western Pacific region has persisted with research focused on the economic drivers of wellbeing in relation to heritage tourism (see Table 3). This persistence may be explained by the relative lack of participation (either as researchers or stakeholders) by local communities in any of the studies included in this review (see Table 4). Indeed, the Western Pacific had the lowest occurrence of community participation and/or consultation in establishing indicators of wellbeing and health and/or opinions about the role of tourism in promoting these.

On the contrary, while seemingly demonstrating the second highest proportion of exclusionary research methods as discussed above, South-East Asia remains the only region where any attempts were made to ensure community members were invited to design and co-lead research (see Table 4). Nonetheless, meaningful modes of participation in this region were found to be more closely in line with the deficits found in Africa, Europe, and the Americas. This lack of approaches aimed at including affected communities as researchers in all but one instance in South-East Asia is an important research gap in tourism studies’ engagement with health and wellbeing debates.

Importantly, this failure to adequately engage with affected communities is at odds with the depth of research emanating from a range of health disciplines, such as disability studies, occupational therapy, public health, and midwifery, where the slogan ‘nothing about us without us’, which emerged in the 1980s, remains prominent. Coupled with a lack of focus on cultural determinants of health, this lack of participation and community direction strongly indicates that research studies are being approached with an *a priori* notion about what ‘wellbeing’ means to local communities, and risks limiting the relevance and accuracy of the research that is being undertaken. Problematically, therefore, there is a tendency to envisage a ‘package’ of wellbeing and health benefits that tourism can potentially bring to a community (regardless of cultural background), with research focusing on identifying the presence or absence of elements of this assumed, overarching ‘package’.

Interestingly, along with the paucity of full and meaningful collaboration with local community hosts in tourism research, there were no instances across the systematic review where a longitudinal approach was adopted. This observation reinforces the point that long-term, collaborative explorations of culturally specific concepts including such things as ‘welfare’, ‘benefit’, ‘healthfulness’ and ‘flourishing’, or combinations of these, are lacking across all regions. To bring tourism research more in line with broader debates and international policy directions about wellbeing, it is important for future research that the qualities of health and wellbeing in a particular cultural setting are investigated as a starting point, and culturally suitable approaches are designed (with local researchers) to best examine the effects of tourism on these contingent notions of wellbeing.

Importantly, a lack of longitudinal research will lead to a gap in our understanding about whether the negative impacts of tourism increase or compound over time. Adopting these ethnographies of health and wellbeing hinges upon long-term community partnerships that will serve to redress a research gap into the longevity of heritage tourism impacts. Furthermore, of those papers that asked local community members about their perceptions of heritage tourism across all regions, a common finding was the desire for greater decision-making and management of the enterprises as stakeholders. It seems ironic, therefore, that research into heritage tourism perceptions itself commonly invites the bare minimum of collaboration to establish the parameters of that research.

In a small number of papers that invited community opinions, local stakeholders considered that the tourism ‘benefits package’ myth should be dispelled, and that responsible tourism development should only happen as part of a wider suite of livelihood options, such as agriculture, so that economic diversity is maintained. Such a multi-livelihood framework would also promote the accessibility of benefits for more of the community, and this poses a significant new direction for tourism research. For example, an outcome of the review was the observation that infrastructure development is often directed towards privileged tourism livelihood options [150], but a more holistic framework would distribute these sorts of benefits to also co-develop other livelihoods.

Although there is a clear interest in understanding the relationship between heritage, tourism, health and wellbeing, future research that explores the intersections of heritage tourism with multiple health domains, in particular social and cultural domains, is critical. Indeed, the frequency with which the negative impacts of heritage tourism were reported in the small number of studies that engaged local community participants suggests that studies co-designed with community participants are a necessary future direction in order for academics, policymakers and professionals working in the field of heritage tourism to more adequately address the scarce knowledge about its socio-cultural impacts. The accepted importance of community researchers in cognate fields underscores that the knowledge, presence and skills of affected communities are vital and points to the need for similar studies in heritage tourism.

## Conclusions

There are five main findings of this systematic review, each of which is a critical gap in research that should be addressed to support the health and wellbeing in local communities at tourism destinations. Firstly, whilst one of the primary findings of this systematic review was the increase in employment opportunities resulting from tourism, this disclosure arose because of a strong–in many cases, exclusive–methodological focus on economic indicators of health and wellbeing. Such research reveals that heritage tourism may significantly reduce poverty and may be used as a poverty-reducing strategy in low-income countries. However, the assumption underlying this focus on the economic benefits of tourism for health and wellbeing is that economic benefits are a proxy for other determinants of health, e.g., cultural, social, environmental, etc., which are otherwise less systematically explored. In particular, the ways in which combinations of environmental, social, cultural, *and* economic determinants on wellbeing interact is an area requires considerable future research.

Secondly, whilst economic drivers of wellbeing were the most common area of research across all regions, the impacts of tourism on *cultural* wellbeing were the least explored. Moreover, in many publications culture was entirely omitted. This is perhaps one of the most troubling outcomes of this systematic review, because in the relatively small number of papers that did investigate the cultural impacts of tourism, many reported traumatising consequences for local communities, the documentation of which would not be recorded in the majority of papers where cultural wellbeing was absent. Tourism’s profoundly damaging consequences included reports of a rise in ethnoreligious violence, loss of ancestral land and the threat of forced relocation, not to mentioned extensive reports of cultural atrophy.

Linked to this lack of understanding about the cultural impacts of tourism on wellbeing, the third finding of this review is that there are far fewer studies that incorporate qualitative data, more suited to document intangible cultural changes, whether positive or negative. Furthermore, more longitudinal research is also needed to address the subtle impacts of tourism acting over longer timescales. The systematic review revealed a lack of understanding about how both the negative and positive outcomes of heritage tourism change over time, whether by increasing, ameliorating, or compounding.

The fourth finding of this research is that, to a degree and in certain regions of the world, research *is* responding to international policy. This review has illustrated that, historically, Africa, Europe and the Americas prioritised research that measured the economic effects of tourism on health and wellbeing. However, after 2019 a shift occurred towards a growing but still under-represented interest in social-cultural wellbeing. We propose that this shift aligns with recommendations from UNESCO’s 2019 *Operational Guidelines for the Implementation of the World Heritage Convention* [148] and the 2020 *Operational Directives for UNESCO’s Convention for the Safeguarding of Intangible Cultural Heritage* [149]. The exception to this shift is the Western Pacific region, where the economic impacts of tourism are increasingly prioritised as the main indicator of wellbeing. Given the overall efficacy of policy for steering towards ethical and culturally-grounded evaluations of the impacts of tourism, we would urge heritage policymakers to take account of our recommendations (Table 2).

The policy implications emerging from this review are the fifth finding and can be distilled into a few key propositions. There is a need for meaningful decolonising approaches to heritage tourism. More than half of the negative consequences of heritage tourism for health and wellbeing could be mitigated with policy guidance, contingent cultural protocols and anti-colonial methods that foreground the rights of local (including Indigenous) communities to design, govern, lead, and establish the terms of tourism in their local area. Although ‘participation’ has become a popular term that invokes an idea of power symmetries in tourism enterprises, it is clear from this systematic review that the term leaves too much latitude for the creep of poor-practice [151] that ultimately erodes community autonomy and self-determination. Participation is not enough if it means that there is scope for governments and foreign investors to superficially engage with community wellbeing needs and concerns.

Furthermore, calls for ‘capacity-building’ that effectively re-engineer the knowledges of local communities are fundamentally problematic because they presuppose a missing competency or knowledge. This is at odds with impassioned anti-colonial advocacy [152] which recognises that communities hold a range of knowledges and cultural assets that they may, and should be legally protected to, deploy (or not) as a culturally-suitable foundation that steers the design of locally-governed tourism enterprises. In short, to maximise and extend the benefits of heritage tourism and address major social determinants of health, host communities’ presence in heritage tourism governance, decision making processes, and control of and access to the resultant community resources and programs must be a priority. Future policymakers are encouraged to make guidance more explicit, enforceable and provision avenues for feedback from local communities that offers the protections of transparency. It is also imperative that researchers involve and empower local community groups as part of studies conducted in relation to their health and wellbeing. If current practices remain unchanged, the primary benefit of tourism could easily be rendered inaccessible through lack of education and/or appropriate training which was frequently identified as a barrier to community participation.

## Supporting information

S1 Checklist PRISMA 2009 checklist. (DOCX)
